# Emerging Argonaute-based food safety detection: from amplification to amplification-free manner

**DOI:** 10.3389/fmicb.2026.1931646

**Published:** 2026-09-10

**Authors:** Wen Kang, Yaru Li, Lu Zhao, Long Ma, Yunfeng Bai, Feng Feng

**Affiliations:** 1 School of Chemistry and Chemical Engineering, Shanxi Provincial Key Laboratory of Chemical Biosensing, Shanxi Datong University, Datong, China; 2 School of Agriculture and Life Science, Shanxi Datong University, Datong, China; 3 State Key Laboratory of Food Nutrition and Safety, College of Biotechnology, Tianjin University of Science and Technology, Tianjin, China

**Keywords:** amplification-free detection, Argonaute, biosensing, food safety, isothermal amplification

## Abstract

Food safety has attracted extensive global attention, making the development of efficient and rapid detection methods essential for food safety risk control. Argonaute-based biosensing has emerged as a promising tool for food safety analysis owing to its unique advantages, including high recognition specificity, low constraints on target sequences, stable and cost-effective guides, and ease of multiplex detection. This review summarized recent progress in Argonaute-based biosensing from amplification to amplification-free manner within the field of pathogenic bacteria, viruses, toxins, etc. It begins with an overview of current nucleic acid detection methods and programmable nucleases. Then, the structure and working principle of Argonaute were briefly introduced. Subsequently, the application of Argonaute-based food safety detection was systematically reviewed according to amplification and amplification-free manners. followed by current challenges and future perspectives. Argonaute provides reliable technical support for food safety monitoring and contributes to the high-quality development of the food industry.

## 1 Introduction

As human progress and the development of the economy and food industry continue, food safety directly impacts consumer health and wellbeing, as well as societal stability. At present, foodborne illnesses caused by chemical hazards are gradually decreasing, while those caused by microbial pathogens are becoming the pre-dominant concern (Yu et al., 2022). Latest statistics from the World Health Organization reveal that roughly 600 million individuals contract illnesses annually due to consuming contaminated food, leading to approximately 420,000 fatalities. Notably, over 70% of these foodborne diseases are caused by pathogenic bacterial infections (Zhang et al., 2025c). Annually, foodborne illnesses may infect approximately 7.69% of the global population and account for about 7.5% of worldwide deaths. Since the early 21st century, foodborne pathogenic bacteria have become a significant factor affecting public health (Li et al., 2025d). These incidents also lead to immeasurable economic losses and substantial damage to the reputation of the food industry. Therefore, ensuring food safety is vital for public health and the economic growth of the food sector. Foodborne pathogens often trigger outbreaks of foodborne diseases, with symptoms including vomiting, nausea, and diarrhea (Jiao et al., 2026). Typical foodborne pathogens include *Salmonella typhimurium* (*S. typhimurium*), *Staphylococcus aureu*s (*S. aureus*), *Vibrio parahaemolyticus, Listeria monocytogenes* (*L. monocytogenes*), and *Escherichia coli*. In recent years, incidents such as group foodborne diseases caused by excessive microorganisms, chronic health damage caused by pesticide and veterinary drug residues, and illegal additives have occurred frequently, fully confirming the core consensus in the field of food safety. More importantly, it is of strategic importance to implement accurate, efficient, and timely detection methods for monitoring and early warning of foodborne pathogens in food products for reducing the damage caused by food contamination and safeguarding public health security (Wei et al., 2023).

The technical proficiency in food safety detection directly dictates our capacity to prevent and control food safety risks, while the precise presentation of each test datum serves as a robust commitment to safeguarding public health. At present, methods to address threats from food safety primarily rely on culture techniques, immunological assays, instrumental analysis, and molecular biology technologies (Li et al., 2022c; Wang et al., 2025a). Nevertheless, these methods exhibit notable limitations in fulfilling the requirements for rapid, sensitive, straightforward, cost-effective, and on-site detection. Traditional culture methods are time-consuming, taking 1–3 days, labor-intensive, and ill-suited for rapid screening. Immunological methods, exemplified by the enzyme-linked immunosorbent assay (ELISA), offer enhanced speed, typically requiring several hours, yet are prone to interference from complex food matrices, potentially leading to false-positive outcomes. Furthermore, these techniques often involve multiple steps, rely on highly specific antibodies, and pose challenges for multiplex detection adaptation (Wang et al., 2020). Instrumental analysis methods, such as high-performance liquid chromatography (HPLC) and liquid chromatography-tandem mass spectrometry (LC-MS), facilitate precise and multiplex detection of food safety contaminants (Li et al., 2023c, 2025a). However, their reliance on expensive equipment and specialized personnel limits practical application, especially in field settings. Molecular biology techniques remain the bedrock of most molecular diagnostics. Nonetheless, the application of these methods is constrained by the necessity for specialized equipment, such as thermal cyclers, and technical expertise. Isothermal amplification techniques (IAT) offer advantages including simplicity, rapidity, high sensitivity, and suitability for on-site detection, enabling ultra-sensitive nucleic acid detection without the need for thermal cycling equipment. Signal readout for molecular detection is typically achieved through colorimetric, lateral flow strips, or fluorescent indicators. Currently, a variety of isothermal amplification techniques have been developed, including recombinase polymerase amplification (RPA), loop-mediated isothermal amplification (LAMP), rolling circle amplification (RCA), among others (Xia et al., 2022; Qiao et al., 2023). However, it is important to note that isothermal amplification methods are susceptible to issues such as primer mispairing or template-independent amplification, which can result in false-positive results. Since target nucleic acids in clinical samples are often present at very low levels, pre-amplification is used to increase target concentration before detection. Nevertheless, this process can prolong detection time, elevate the risk of aerosol contamination, and complicate operations, thereby limiting its suitability for point-of-care testing (POCT) (Ye et al., 2024).

Programmable nucleases are revolutionary tools in the field of modern biotechnology, which can accurately identify target nucleic acid sequences through guided nucleic acids (RNA or DNA) and perform specific cleavage, bringing breakthrough progress to gene editing, nucleic acid detection, synthetic biology, and other fields (Li et al., 2022a; Qin et al., 2022; Li et al., 2023b). Among them, the CRISPR/Cas system and Argonaute are the two most representative types of programmable nucleases. CRISPR/Cas and Argonaute have significant differences in origin, working mechanism, core characteristics, and application scenarios while also forming functional complementarity, jointly promoting the rapid development of molecular biology research and biotechnology industrialization. The CRISPR (Clustered Regularly Interspaced Short Palindromic Repeats)/Cas system is an adaptive immune system developed by bacteria and archaea over a long period of evolution to resist the invasion of foreign nucleic acids such as bacteriophages and plasmids. The application of CRISPR/Cas in detection mainly utilized the “collateral cleavage activity” of Cas12a (non-specific cleavage of single-stranded DNA reporter molecules after cutting target DNA) or the “RNA incidental cleavage activity” of Cas13 to develop highly sensitive and specific nucleic acid detection technologies (Abudayyeh et al., 2016; Chen et al., 2018; Ackerman et al., 2020). Argonaute is a class of conserved nucleases widely present in prokaryotes and eukaryotes (Hegge et al., 2019). The working mechanism of Argonaute followed the principle of “guided nucleic acid-mediated target recognition and cleavage” (Swarts et al., 2014). However, there are also several distinctions between them. First, the guide nucleic acids: CRISPR/Cas systems employ crRNA, whereas Argonaute proteins use shorter 5′-phosphorylated or 5′-hydroxylated DNA/RNA guides. Second, sequence-dependent constraints: CRISPR/Cas-based assays generally require PAM (Protospacer Adjacent Motif). This PAM requirement leads to low universality and limits the available loci for CRISPR RNA (crRNA) design (Ng et al., 2025) By contrast, Argonaute mediates sequence-dependent target cleavage (Kuzmenko et al., 2020; Liu et al., 2021). Liu et al. (2022) developed a novel method of the on-site detection for Salmonella in food by combining the CRISPR/ Cas12a system with RPA. They added the Cas system, which contained the Cas12a enzyme, crRNA, and FQ-probe, directly to the cover of the tube in which the RPA reaction mixture was covered with 15 L of mineral oil. The detection was carried out in one tube and the generated fluores cence was analyzed using a portable blue-light transilluminator. Target recognition by crRNA, activation of Cas12a-specific cleavage, and subsequent fluorescence signal generation through trans-cleavage activity all require the presence of a conserved PAM in the target DNA sequence. Lin et al. (2024) established a method combining RPA and *Pf* Ago for fast *Salmonella* spp. testing. The invA gene of *Salmonella* spp. was amplified by RPA, followed by *Pf* Ago- based target sequence cleavage, which could be observed by a fluorescence reader or the naked eye. The former, Cas12a, is constrained by PAM sequences. Target and amplicon design must match PAM sites, which somewhat limits the selection of primers and target sites. In contrast, *Pf* Ago does not rely on PAM sequences, allowing greater flexibility in designing targets and amplification primers. This is one major advantage of Argonaute over the CRISPR-Cas system. Third, cleavage activity: CRISPR/Cas exhibits both cis- and trans-cleavage; trans-cleavage, unique to Cas12a and Cas13a, is exploited as a biocatalytic signal transducer and amplifier to improve detection sensitivity. In addition, Cas enzymes tolerate base mismatches close to the PAM sequence well, yet they frequently fail to detect single-point mutations at central positions. Therefore, they are not ideal for accurate genotyping of SNPs and drug-resistance sites. By comparison, Argonaute provides superior single-base resolution with lower mutation tolerance in detection. A mismatched base between gDNA and target will substantially suppress its cleavage activity. Such single-base discrimination is highly desirable for SNP detection, which aims to distinguish single-nucleotide alterations. Unlike equipment-hungry conventional SNP detection techniques, *Pf* Ago couples programmable guide-DNA-directed cleavage with strict base-pairing requirements. Tang et al. (2024) combined *Pf* Ago with isothermal RPA amplification to detect the long-COVID-associated rs17713054 G>A SNP in the LZTFL1 gene. RPA-amplified wild-type or mutant templates trigger cascaded *Pf* Ago cleavage reactions, yielding allele-specific fluorescence outputs from molecular beacons. This isothermal assay achieves single-copy sensitivity. The CRISPR/Cas system has been widely used in gene editing, disease treatment, and other fields, while the Argonaute provides a new solution to the off-target effects and temperature limitations of the CRISPR/Cas system (Gootenberg et al., 2017; Fozouni et al., 2021; Li et al., 2025d). With the in-depth analysis and optimization of the molecular mechanisms of two types of systems, programmable nucleases could show broader application prospects in fields such as food safety and medical diagnosis, continuously promoting the revolutionary development of biotechnology.

**Figure 1:**
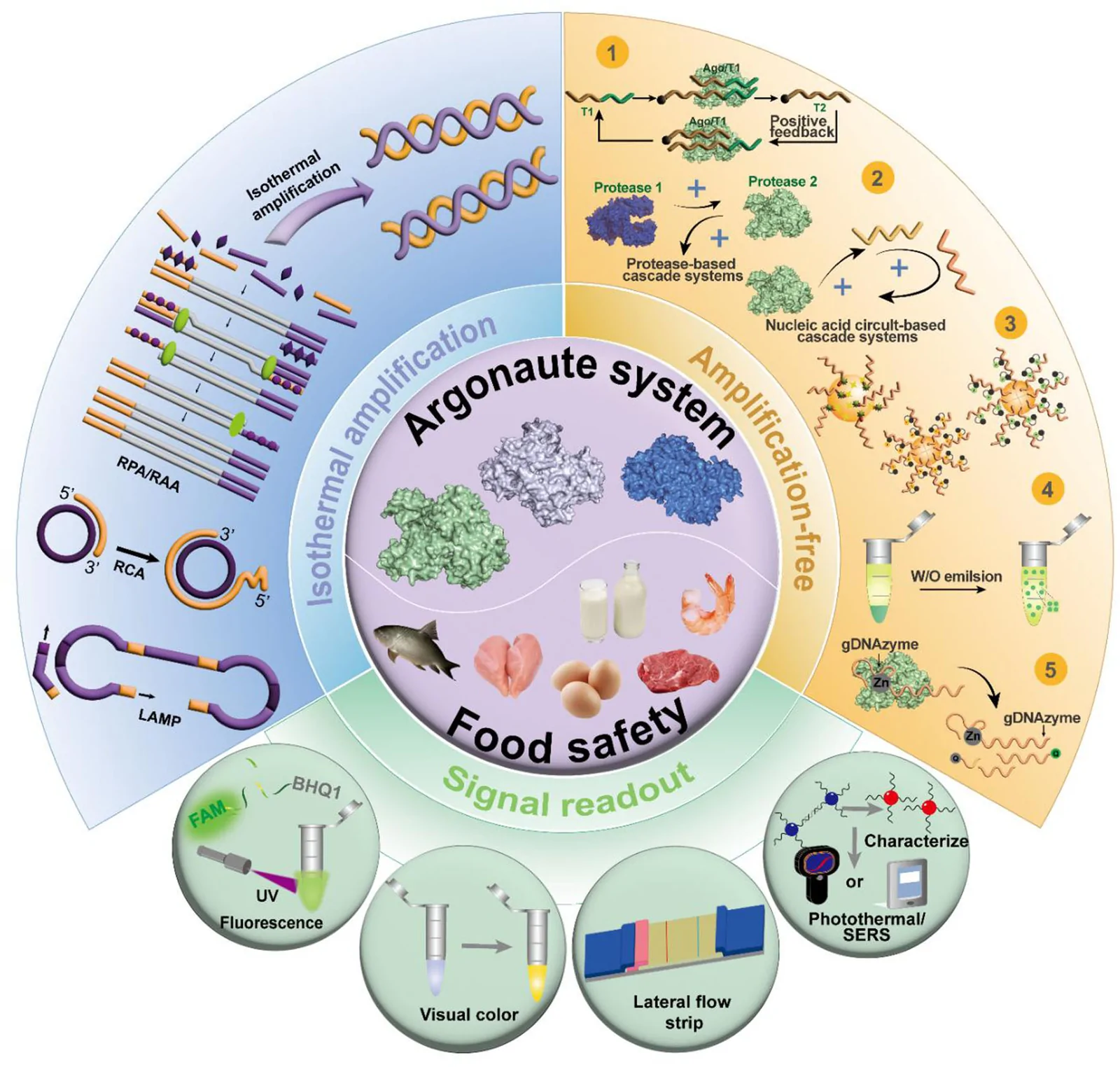
The overview of emerging Argonaute-based food safety detection.

Li et al. (2024e) summarized the current progress of Argonaute-based nucleic acid detection. In addition, the same team presented their insights and perspectives on Argonaute-based foodborne pathogen detection (Li et al., 2025d). Yang et al. (2023) reviewed the development of CRISPR/Cas- and Argonaute-based electrochemical biosensors for pathogen. Li et al. (2024d) provided a CRISPR/Cas- and Argonaute-powered lateral flow assay for pathogen detection. In 2025, the same team presented a comprehensive review and synthesis of the advancements in CRISPR/Cas systems and Argonaute-based strategies for multiplexed detection Li et al., (2025c). A meticulous examination of the current review literature uncovers a significant absence of systematic and all-encompassing summaries centered on Argonaute-based food safety detection strategies, encompassing both amplification-based and amplification-free methodologies. To address this deficiency, this review underscores the innovative and multifaceted utilization of Argonaute proteins in crafting food safety detection techniques. We further accentuate the progress, novelty, and practical applicability of cutting-edge technologies spanning from amplification-dependent to amplification-free systems, along with their corresponding challenges and future outlooks (Figure 1). Overall, this review makes a timely and meaningful contribution to bridging this knowledge gap. It is expected to enhance researchers' comprehension of Argonaute and sophisticated detection methods, while offering valuable insights and guidance for the creation of new detection platforms.

## 2 Classification, structure, working principle and advantage of Argonaute

Argonaute proteins are a class of programmable nucleases widely present in both eukaryotic and prokaryotic organisms. In 1998, Bohmert and colleagues first mentioned Argonaute in their study describing Arabidopsis mutants (Bohmert et al., 1998). Argonaute proteins are classified into eAgo (eukaryotic Argonaute) and pAgo (prokaryotic Argonaute). eAgos mainly play a crucial role in the RNA interference pathway, which could utilize small RNAs as guides to recognize or cleave complementary RNA sequences. pAgos not only could function as endonucleases *in vitro* but may also participate in cellular defense mechanisms *in vivo* (Wang et al., 2008). The majority of pAgos and eAgos exhibit a high degree of structural homology. However, they differ in the guide and target preferences. pAgos originate from diverse prokaryotic species, some of which could utilize DNA guides to target DNA or RNA substrates, while a minority employ RNA guides. eAgos typically bind to RNA targets complementary to their guides, whereas the majority of pAgos associate with DNA targets that are complementary to their guides (Sheng et al., 2014).

The core domains of all Argonaute proteins are arranged from the N-terminus to the C-terminus as follows: N-terminal domain, PAZ domain, MID domain, PIWI domain, and some members also include a C-terminus extension. The N-terminal domain participates in the dissociation of double-stranded RNA and facilitates RNA target cleavage. In the ternary complex structure formed after *Thermus thermophilus* (*Tt*Ago) binds to the guide and target strands, the N-terminal domain is observed to prevent the pairing of the target with the guide beyond the 16th nucleotide of the guide (Wang et al., 2009; Kwak and Tomari, 2012). The PAZ domain features an SH3-like barrel fold binding pocket that facilitates interaction with the 3′ end of the guide RNA. In the crystal structure of the binary complex formed between *Tt*Ago and the guide, the phosphate and hydroxyl groups for 3′ end of the guide coordinate with amino acid residues within the PAZ domain. The guide and Argonaute seed regions are pre-assembled into an A-form helical conformation stabilized by a phosphate backbone. The two nucleotides of the seed region are solvent-exposed to promote Watson-Crick base pairing between the guide and target. The remaining portion of the guide RNA passes through the protein's central channel, where it is sandwiched between two lobes and extensively hydrogen-bonded to the hydrophilic channel of the protein. In the ternary complex involving *Tt*Ago, the guide and the target with varying degrees of complementarity, base pairing beyond the 15th nucleotide of the guide (g15) triggers the release of the guide's 3′ end from the PAZ domain (Kwak and Tomari, 2012; Li et al., 2025a). In pAgo proteins, the PAZ domain anchors the 3′ end of the guide strand during target recognition. When the pAgo/guide complex locates a complementary sequence on the target strand, the 3′ end is released, allowing complete base pairing between the guide and substrate, thereby activating the endonuclease activity of the PIWI domain. This domain contains a metal-dependent RNase H-like catalytic site that cleaves the phosphodiester backbone of the complementary nucleic acid substrate; in the case of double-stranded substrates, only the strand complementary to the guide is cleaved. Finally, the N domain functions as a wedge, facilitating the release of the target DNA. The MID domain stacks the g2-g10 nucleotides of the guide strand (positions 2 to 10) into a helical conformation, with the g2-g6 region exposed to the solvent, facilitating sequence-specific recognition and binding of the target. The MID domains contain four conserved amino acid residues that coordinate with the5′ phosphate group of the guide (Lisitskaya et al., 2018). This explains why they require the guide strand containing a 5′-phosphate group (Kaya et al., 2016). The PIWI domain constitutes the catalytic core of Argonaute proteins (Liu et al., 2004). The PIWI domain undergoes conformational transitions between two states: a cleavage-compatible state and a cleavage-incompatible state. During the transition, the glutamic acid residue within the catalytic tetrad shifts from an “external” to an “internal” conformation, forming a complete catalytic center. This conformational change requires pairing between g2 and g16 and the target (Sheng et al., 2014).

*Pf* Ago, *Tt*Ago, and *Cb*Ago are prokaryotic Argonaute (pAgo) proteins that mediate immune defense against foreign nucleic acids. All of them utilize 5′-phosphorylated DNA guides to cleave target nucleic acids in a PAM-independent manner, yet they exhibit substantial differences in temperature adaptability, enzymatic properties, and application scenarios. Derived from *Pyrococcus furiosus, Pf* Ago represents the most well-structurally characterized and extensively applied pAgo for in-vitro use. It displays robust cleavage activity exclusively at high temperatures of 87–99 °C and is nearly inactive at room temperature. *Pf* Ago requires 5′-phosphorylated DNA guides longer than 14 nt to cleave both single and double-stranded DNA. Its high-temperature reaction can disrupt DNA secondary structures, enabling the generation of customizable sticky ends as artificial restriction endonucleases and expanding the target-design flexibility for biosensing. Nevertheless, such stringent high-temperature requirements impose higher demands on experimental equipment. Derived from *Thermus thermophilus, Tt*Ago has an optimal temperature range of 65–85 °C. It employs DNA guides to cleave both DNA and RNA targets. Under native physiological conditions, *Tt*Ago assembles a ternary complex with 13–25 nt small interfering DNA (siDNA) to specifically cleave invading nucleic acids. *Tt*Ago is widely utilized for precise nucleic acid cleavage, mutation detection, and auxiliary screening coupled with isothermal amplification; nevertheless, its thermophilic property also restricts its application in point-of-care rapid detection. Derived from *Clostridium butyricum, Cb*Ago exhibits an optimal temperature range of 30–60 °C and can precisely cleave double-stranded DNA targets at room temperature. Featuring high activity, high specificity and no PAM-motif preference, *Cb*Ago effectively addresses the high-equipment requirements of the two pAgos. It is compatible with point-of-care testing (POCT) and integration with multiple sensing platforms, with the main limitation being its inability to disrupt DNA secondary structures, which renders it less efficient for complex nucleic acid samples. Overall, *Pf* Ago is preferable for in-vitro molecular manipulations that require complete disruption of high-order DNA structures; *Tt*Ago is suitable for the detection and cleavage of diverse target nucleic acids under moderate-to-high temperature conditions, whereas *Cb*Ago serves as a more feasible pAgo tool for room-temperature, portable on-site biosensing.

The core operating principle underlying Argonaute-mediated detection relies on three key features: guide dependent target recognition, nuclease activity (cleavage of targets or reporter molecules), and structural conservation (adaptation to *in vitro* reactions). The essence is to modify “guide target recognition cleavage” molecular mechanism of Argonaute *in vitro*, converting it into detectable signals (such as fluorescence, colorimetry, etc.). Argonaute-based detection not only retained the specificity of biomolecules, but also adapted the practicality of biosensing technology through engineering design.

The core advantages of Argonaute are as follows. First, guide diversity: DNA guides can be used (e.g., *Tt*Ago can utilize 5′-phosphorylated ssDNA as a guide), thus avoiding the degradation issues associated with RNA guides. Moreover, DNA guide design is more flexible. Second, temperature adaptability: some prokaryotic Argonaute proteins originate from thermophilic bacteria and function efficiently under high temperatures, thereby reducing non-specific binding. Third, high cleavage specificity: the base-pairing requirement between the guide and the target is stringent, making off-target effects less likely. Fourth, convenience for multiplexed detection: based on sequence-specific binding and cleavage, Argonaute can recognize multiple targets simultaneously by designing complementary guides for different targets. Multiplexed signal-reporting systems can also be constructed, where different targets are coupled with distinct signal reporters to enable simultaneous discrimination of multiple signals. The unique properties of Argonaute make it a valuable complement to the CRISPR/Cas system. It can be applied to address food safety detection issues and establish a reliable safeguard for food safety, serving as an emerging technical tool to empower the advancement of food safety assurance.

## 3 Application

This section systematically summarizes and reviews the latest advances in nucleic acid detection strategies based on Ago proteins. Representative Ago-enabled biosensors for nucleic acid target recognition are summarized in Table 1. Such Ago-based analytical technologies hold great promise for future market applications focused on analyte detection.

**Table 1:** Characteristics of Argonaute-based food safety detection technologies.

Isothermal amplification method	Agronaute	Method	Target	Readout	LoD (sensitivity)	Detection time	**Ref**.
RPA	*Pf*Ago	RPA-*Pf*Ago	*Salmonella*	Fluorescence	1.05 × 10 CFU/ml	40 min	Lin et al., 2024
RPA	*Pf*Ago	RPA-*Pf*Ago	MRSA, *S. aureus*	Fluorescence	10^2^ copies/μl	55 min	Chen et al., 2024
RPA	*Cb*Ago	RPA-*Cb*Ago	*S. Typhimurium, L. monocytogenes*	Microdroplet area and fluorescence	1 CFU/ml	45 min	Wen et al., 2024
RPA	*Pf*Ago	RPA-*Pf*Ago	MS	Super-interpreted under UV and blue light	2 copies/ml	40 min	Zhao et al., 2024d
RAA	*Pf*Ago	RAA-*Pf*Ago	*S. aureus*	Fluorescence, LFS	1 CFU/ml	1.5 h	Li et al., 2024b
LAMP	*Pf*Ago	STAR	MRSA	Fluorescence	10 CFU/ml	65 min	Kou et al., 2024
LAMP	*Pf*Ago	Argonaute-DNAzyme	MRSA	Fluorescence	1 CFU/ml	\	Li et al., 2024c
LAMP	*Tt*Ago	ASAP	*S. aureus*	Fluorescence	1 CFU/ml	17 min	Zhao et al., 2024c
LAMP	*Pf*Ago	LAMP-*Pf*Ago	*L. monocytogenes*	LFS	1.8 × 10^2^ copies	80 min	Yu et al., 2024b
LAMP	*Pf*Ago	*Pf*Ago-LFB	*S. typhimurium*	Fluorescence, LFS	1 CFU/ml	45 min	Qiao et al., 2024
LAMP	*Pf*Ago	*Pf*Ago-hemins@ZIF-90	*S. typhimurium*	Colorimetry	1 CFU/ml	60 min	Zhang et al., 2025b
LAMP	*Pf*Ago	LAMP-Aurora DNAzyme- *Pf*Ago	S. *typhimurium*	Fluorescence	1 CFU/ml	60 min	Zhang et al., 2025a
LAMP	*Pf*Ago	PASS	*S. typhimurium*	SERS	1 CFU/ml	60 min	Lu et al., 2025
LAMP	*Pf*Ago	*Pf*Ago-rLFTS	*L. Monocytogenes* (*hly* and *lde* genes)	Colorimetry, Fluorescence	1 CFU/ml	45 min	Tang et al., 2025
RPA	*Pf*Ago	DIDCi	White spot syndrome virus, Vibrio bacteria	Fluorescence	10^2^ gene copies	105 min	Chen et al., 2025
RPA	*Pf*Ago	RPA-*Pf*Ago	Goose parvovirus	Fluorescence	10^2^ GPV DNA copies	\	Liu et al., 2024
RPA	*Pf*Ago	RPA-*Pf*Ago	White spot syndrome virus	Fluorescence	single copy	75 min	Wang et al., 2023
RPA	*Pf*Ago	RT-RPA-*Pf*Ago	Rice ragged stunt virus, rice grassy stunt virus, rice black streaked dwarf virus	Fluorescence	3.13 - 5.13 copies/μl	\	Liu et al., 2023
RAA	*Pf*Ago	one-pot RT-RAA-*Pf*Ago, one-pot RT-RAA-*Pf*Ago-LFD	Porcine deltacoronavirus	Fluorescence	60 copies/μl	\	Zhao et al., 2024c
RAA	*Pf*Ago	REPD	African swine fever	Fluorescence	single copy	80 min	Zhao et al., 2024b
LAMP	*Pf*Ago	RT-LAMP-*Pf*Ago	Porcine epidemic diarrhea virus	Fluorescence	2.4 copies/μl	35 min	Yu et al., 2025
LAMP	*Pf*Ago	LAMP-*Pf*Ago	fowl adenovirus serotype 4	Fluorescence	5 copies	50 min	Yu et al., 2024a
RCA	*Pf*Ago	*Pf*Ago-RCA	Zearalenone	Fluorescence	5.3 pg/ml	15 min	Zhu et al., 2025
\	*Pf*Ago	DECA	*S. typhimurium*	Fluorescence	23 CFU/ml	60 min	Chen et al., 2023a
\	*Pf*Ago	PDAAF	*Salmonella*	Fluorescence	20 CFU/ml	30 min	Wang et al., 2024b
\	*Tt*Ago	ANCA	*Klebsiella pneumoniae*	Fluorescence	1 fM	\	Jang et al., 2023
\	*Cb*Ago	PASS	*L. monocytogenes S. typhimurium S. aureus*	Fluorescence	10^3^ CFU/ml; 10^2^ CFU/ml; 10^2^ CFU/ml	1 h	Wang et al., 2024c
\	*Cb*Ago	\	*S. typhimurium*	Fluorescence	40.5 CFU/ml	\	Zhao et al., 2024a
\	*Cb*Ago	*Cb*Ago -DNAzyme	*S. aureus, E. coli, Salmonella*	Fluorescence	46 CFU/ml, 76 CFU/ml, 75 CFU/ml	\	He et al., 2024
\	*Cb*Ago	\	*S. typhimurium, S. aureus, L. monocytogenes*	Fluorescence	6 CFU/ml	\	Wang et al., 2025b
\	*Cb*Ago	PS-Dot	*Salmonella, L. monocytogenes, S. aureus*	Fluorescence	10^2^ CFU/ml	1.5 h	Wang et al., 2024d
\	*Cb*Ago	MAEA	*E. coli, S. aureus*	Fluorescence	10^2^ CFU/ml	2 h	Lu et al., 2024
\	*Cb*Ago	RCA-*Cb*Ago	MRSA *mecA* and *femA*	Fluorescence	0.63 fM, 0.48 fM	\	Wei et al., 2024
\	*Cb*Ago	*Cb*Ago-PS-TDNA-HCR	*S. aureus*	Fluorescence	10^2^ CFU/ml	1.5 h	Wang et al., 2025a
\	*Cp*Ago	*Cp*Ago-SERS	Cytb, Actb, ND2	SERS	0.497 ng/μl	35 min	Liu et al., 2025
\	*Tt*Ago	(EDC)-Argonaute	fumonisin B1	Fluorescence	0.89 pg/ml	\	Lin et al., 2025
\	*Cb*Ago	TDNA@MNP	AFB1, AFG1, DON, *E. coli, S. aureus, S. typhimurium*	Fluorescence	pg/ml level 10^2^ CFU/ml	1 h	Li et al., 2024a
\	*Cb*Ago	\	mycotoxins AFB1, OTA, DON, PCT and IL-6	Fluorescence	30.05 pg/ml, 2.81 pg/ml, 14.27 pg/ml, 109.50, 5.30 pg/ml	30 min	Li et al., 2025b

### 3.1 Isothermal amplification integrated Argonaute for food safety detection

The integration of Argonaute and isothermal amplification can serve as a “double-checking” for enhancing accuracy, adaptability, sensitivity and dependability of the bioassay method. The cascade reaction involving “amplification plus specific recognition” enables two-stage signal enhancement. Isothermal amplification achieves target enrichment by a factor of 10^6^–10^9^, whereas Argonaute-mediated cleavage or binding further converts target recognition events into readable signals. By using this synergistic strategy, ultra-low abundance targets can be detected, which are difficult to be detected by other technologies alone.

#### 3.1.1 The integration of RPA/RAA and Argonaute

**Figure 2:**
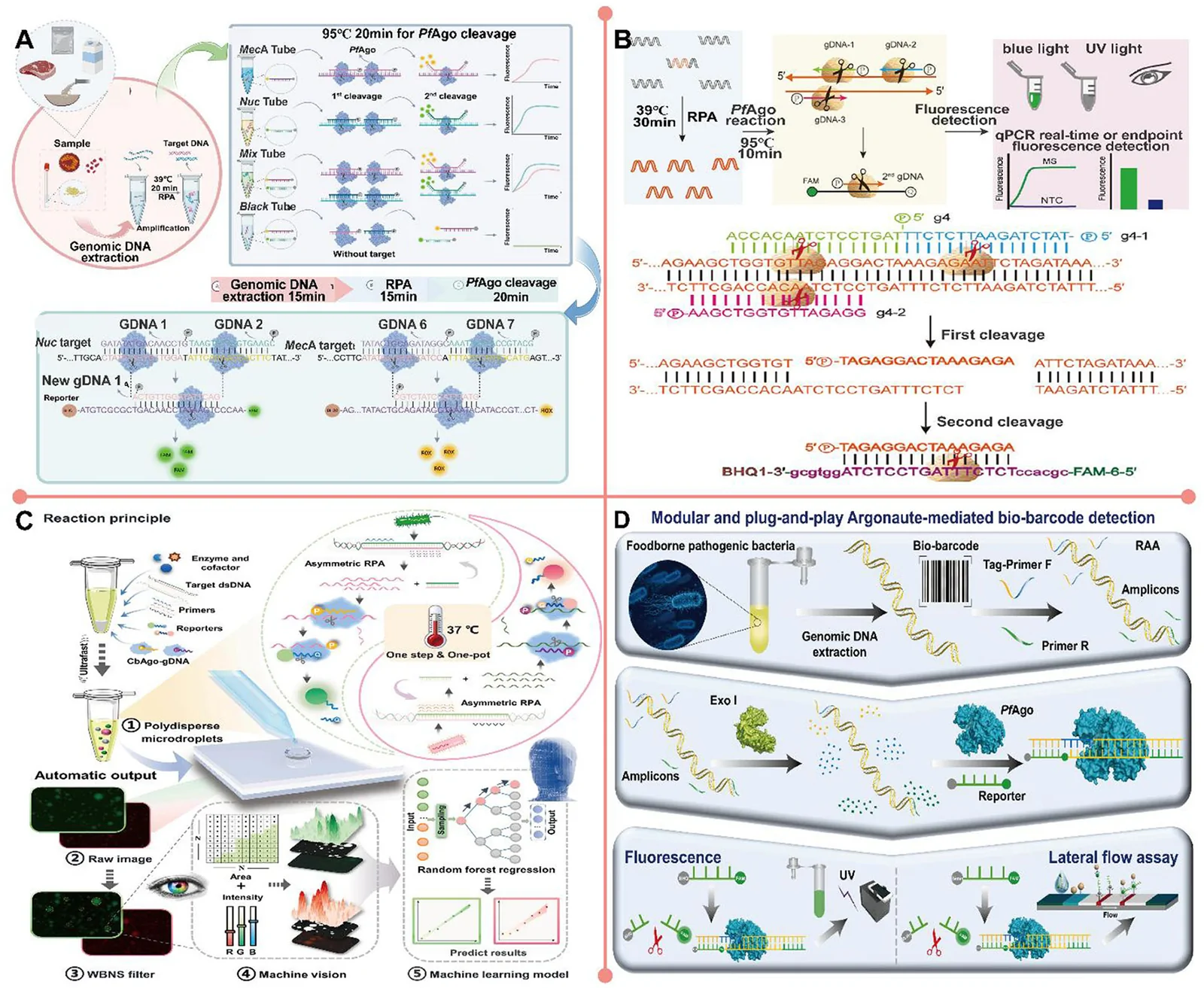
The integration of RPA/RAA and Argonaute for pathogenic bacteria. **(A)** RPA-*Pf* Ago-based MRSA detection (Chen et al., 2024). **(B)** RPA-*Pf* Ago-based MS detection (Zhao et al., 2024d). **(C)** An Argonaute-based digital platform for foodborne pathogen detection (Wen et al., 2024). **(D)** Argonaute-based bio-barcode assay for *S. aureus* (Li et al., 2024b).

RPA relies on key enzymes including recombinases, single-strand binding proteins, and strand-displacing DNA polymerases to achieve rapid nucleic acid amplification under isothermal conditions ranging from 37 to 42 °C. As illustrated in Figure 2A, Lin et al. (2024) developed a highly sensitive and accurate detection method for *Salmonella*. In this system, RPA amplification was followed by target sequence cleavage directed by *Pyrococcus furiosus* Argonaute (*Pf* Ago), yielding a detection limit (LOD) of 10.5 CFU/ml. Chen et al. (2024) constructed an efficient detection system for MRSA by combining RPA with *Pf* Ago-mediated specific cleavage of the *nucA* and *mecA* genes. Similarly, FAM/BHQ1-labeled hairpin molecular probes could emit fluorescence signals after *Pf* Ago-driven dual cleavage (Figure 2B) (Zhao et al., 2024d). *Pf* Ago demonstrates activity at relatively elevated temperatures, with an optimal range reaching up to 87–99.9 °C, which restricts its application under ambient conditions. Thus, Sun's team innovatively expressed and purified a mesophilic *Clostridium butyricum* Ago (*Cb*Ago), which operates efficiently at moderate temperatures (37 °C). *Cb*Ago initially binds to a specifically designed guide nucleic acid to form a functional complex capable of recognizing targets. This complex achieves precise recognition through complementary base pairing between the guide nucleic acid and targets, and single base mismatches significantly reduce binding efficiency. Upon target binding, the nuclease activity of *Cb*Ago is activated, enabling it to cleave targets and generate signals by cleaving reporter molecules or triggering cascade reactions, thereby converting targets into detectable signals. As shown in Figure 2C, *Cb*Ago-medicated molecular detection process combined with intelligent analysis has been established, encompassing three core steps: reaction system construction, signal acquisition, and intelligent analysis (Wen et al., 2024). Specifically, the target, enzyme, cofactor, primer, reporter molecule, *Cb*Ago-gDNA, and other reagents are mixed to generate polydisperse microdroplets through an “ultra-fast” operation, serving as microcavities for the reaction. Target DNA amplification is achieved through asymmetric RPA under the “one-step/single tube” condition at 37 °C. The amplified product generates a signal after being specifically recognized by *Cb*Ago-gDNA. The original output image is denoised and enhanced using a WBNS filter to obtain a clear signal speckle pattern. Machine vision analysis is employed to identify the micro droplet area and brightness of processed images, extract signal features from different channels (R/G/B), and convert visual information into quantitative data. This method utilizes a novel oil-in-water multiphase microdroplet reactor system combined with an intelligent, multivariate image analysis platform based on microdroplet area and fluorescence intensity to enhance signals. It addresses the drawbacks of traditional microdroplet digital nucleic acid platforms, such as the requirement for specialized instrumentation to produce monodisperse microdroplets, costly thermal cyclers, compatible high-temperature resistant reagents, and labor-intensive multi-step procedures, thereby enabling true one-pot digital nucleic acid detection. Moreover, the method facilitates integrated detection of molecular amplification, signal visualization, intelligent quantitative analysis, characterized by easy operation (single tube/isothermal reaction), a high degree of automation, and the combination of machine vision and AI analysis to enhance detection accuracy. The above-mentioned methods are all based on Argonaute-mediated two-step cleavage. Thus, Li et al. (2024b) innovatively proposed a one-step cleavage that combines *Pf* Ago, RAA and LFS for one-tube and onsite detection of *S. aureus*. As illustrated in Figure 2D, the genomic DNA of *S. aureus* is extracted and added to RAA reaction. Notably, forward primers are barcoded with tag sequences. After amplification, these tag sequences are incorporated into the amplified products. Exo I is then added to digest excess primers, preventing the generation of incorrect signals. The amplified products, barcoded with tag sequences, serve as guide DNA to trigger *Pf* Ago for one-step cleavage of FAM-BHQ/biotin labeled reporter, thereby producing detectable signals. The method achieves an LOD of 1 CFU/ml. The superior detection sensitivity of the one-step cleavage system stems from the synergistic effect of multiple mechanisms. This method avoids the drawbacks of the two-step cleavage strategy, including the requirement for pre-synthesis and extra addition of exogenous gDNA, as well as the non-specific degradation and futile consumption of gDNA. RAA amplification endogenously generates tagged amplicons that directly serve as guide DNAs for *Pf* Ago *in situ*, achieving efficient coupling between amplification and Ago-mediated cleavage. Each nascent amplicon produced from RAA-amplified targets can directly activate *Pf* Ago to cleave reporter molecules, enabling amplicons to function in both target recognition and Ago activation and producing a signal cascade amplification effect of “one-round amplification triggering multiple rounds of Ago-dependent reporter cleavage”. Furthermore, exonuclease I (Exo I) incorporated in the system specifically degrades residual unconsumed free tagged forward primers, preventing non-specific cleavage of reporter probes caused by residual primers acting as false guide DNAs. This approach substantially reduces background noise and improves the signal-to-noise ratio for low-copy target detection, while preserving faint valid signals derived from trace genuine targets. Continuously activated *Pf* Ago by endogenous guide DNAs repeatedly cleaves FAM-BHQ-biotin dual-labeled reporter probes. The released FAM-biotin fragments are continuously enriched and accumulated on lateral-flow strips (LFS). Benefiting from the unique cumulative signal readout mode of LFS, faint signals corresponding to trace targets can be efficiently captured, thus enabling accurate detection of targets at extremely low concentrations. Therefore, this method achieves remarkably high sensitivity.

#### 3.1.2 The integration of LAMP and Argonaute

**Figure 3:**
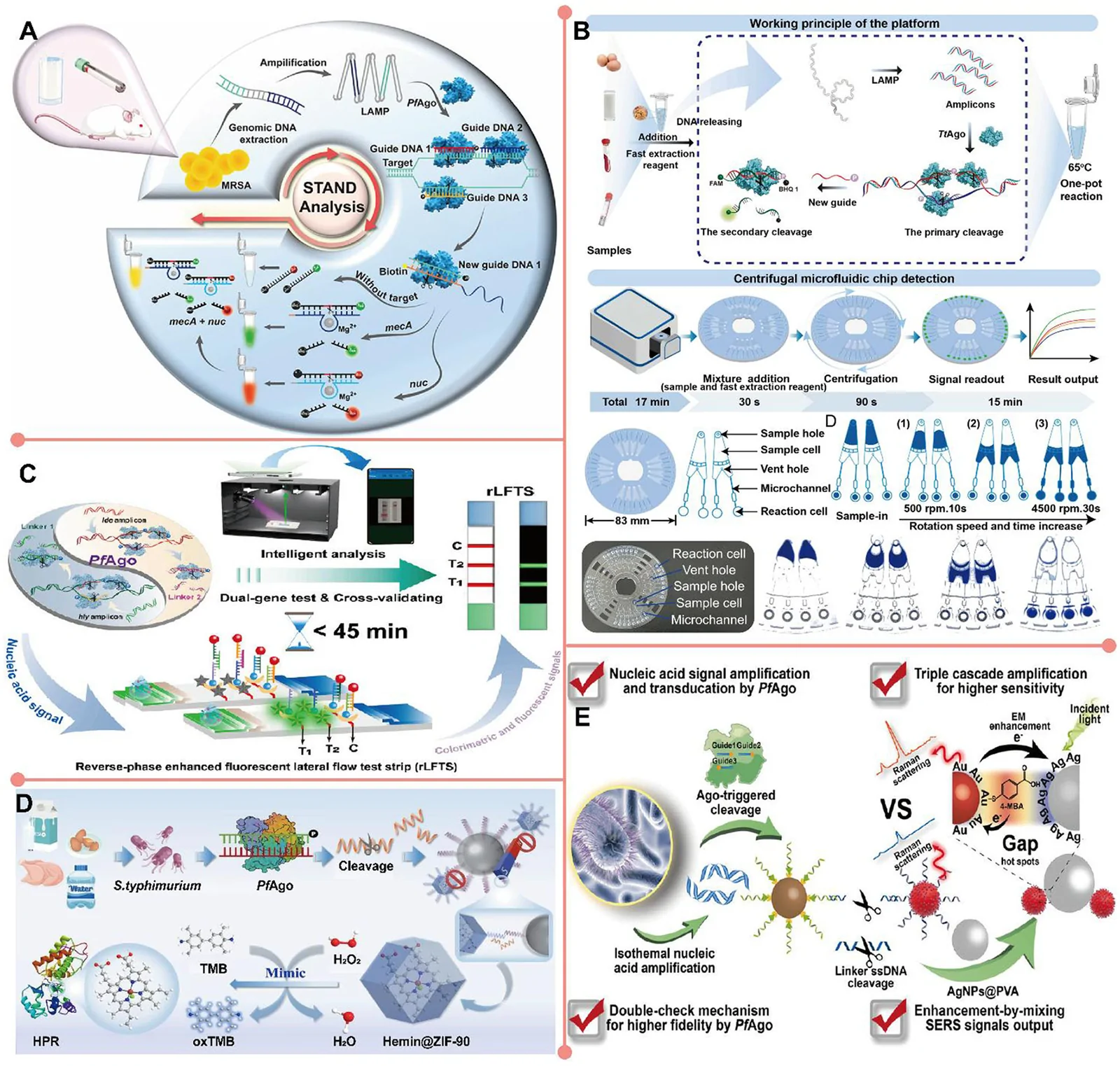
The integration of LAMP and Argonaute for pathogenic bacteria. **(A)** Argonaute-based tandem biosensing for MRSA detection (Li et al., 2024c). **(B)** *Tt*Ago-based centrifugal microfluidic chip reaction biosensor for *S. aureus* diagnostics (Zhao et al., 2024c). **(C)** Argonaute biosensing platform based on Hemin@ZIF-90 nanozyme-modulated signal amplification (Zhang et al., 2025b). **(D)** Argonaute-triggered and reverse-phase enhanced fluorescent lateral flow bioassay for *L. monocytogenes* detection (Tang et al., 2025). **(E)** Argonaute-triggered and nano-SERS-transduced triple cascade amplification strategy (Lu et al., 2025).

LAMP relies on *Bst* DNA polymerase with strand-displacement activity to complete nucleic acid amplification at a constant temperature of 60–65 °C without the thermal-cycling process required by PCR instruments. This technology mainly uses four specific primers to recognize and amplify target sequences, so the amplification step itself exhibits strong target-recognition capability. When LAMP is combined with Ago, it achieves dual-specificity protection through multi-primer screening during amplification and precise recognition by Ago nuclease, which helps reduce the occurrence of false-positive results. When LAMP is coupled with thermostable Argonaute (*Pf* Ago, *Tt*Ago) to construct a detection system, their reaction temperatures are well-matched (LAMP: 60–65 °C; optimal cleavage temperature of *Pf* Ago: 70–75 °C). LAMP amplicons can be directly used for Ago-mediated cleavage without cooling, which facilitates the establishment of time-sequenced one-tube detection systems and reduces the risk of aerosol contamination caused by opening and transferring samples. In contrast, amplification in the RPA/RAA-Argonaute system proceeds only at 37–42 °C, showing a clear temperature difference from the high-temperature cleavage range of thermostable Ago proteins. Additional temperature switching is required for one-tube system development, which increases the difficulty of system optimization (Fan et al., 2025; Sun et al., 2025). Ma and colleagues have developed a portable biosensor utilizing LAMP and *Pf* Ago (Kou et al., 2024). In this system, the specific *mecA* and *nuc* genes are amplified via LAMP. The amplified products are then specifically recognized by *Pf* Ago and generate new guide DNA to cleave reporter molecules labeled with fluorescent and quenching groups. When only the *nuc* gene is present, fluorescence signals are generated in the red emission (ROX channel), indicating the presence of methicillin-sensitive *Staphylococcus aureus* (MSSA). Conversely, the strong green fluorescence signals (FAM channel) are produced only the *mec*A gene, signifying the presence of *mec*A-harboring species, such as *mec*A-positive *S. aureus* strains, *S. epidermidis* and *S. haemolyticus*. When MRSA is present, both mecA and nuc gene amplicons are cleaved by *Pf* Ago, resulting in a discernible yellow signal derived from the fusion of green (FAM) and red (ROX) fluorescence. This dual-channel (0,1) fluorescence pattern provides a straightforward digital coding scheme for MRSA identification. Additionally, a cost-effective 3D-printed reaction chamber and imaging device have been fabricated for rapid detection in POCT. The method achieves a LOD of 10 CFU/ml. Subsequently, the same team integrated DNAzyme into the previously described system to further improve detection sensitivity (Li et al., 2024c). As illustrated in Figure 3A, after *Pf* Ago-based two-step cleavage, the fluorescent probe is replaced by a fluorescent and biotin-labeled DNAzyme, which consists of three parts: biotin, a sequence complementary to the new guide DNA and DNAzyme. When the target is present, *Pf* Ago is activated to cleave the reporter, causing the separation of DNAzyme and biotin. After adding streptavidin-labeled magnetic beads, the supernatant containing the DNAzyme is separated. Once Mg^2+^ is mixed, DNAzyme cleaves the double-labeled fluorescent reporter (FAM-BHQ1 and ROX-BHQ2), leading to fluorescence recovery. The LOD can achieve 1 CFU/ml, which is more sensitive than the original Argonaute-based biosensors by a factor of 1~2 orders of magnitude. The improved sensitivity achieved by DNAzyme integration primarily benefits from the secondary catalytic signal amplification strategy. In the original sensing system, fluorescent signals were generated merely through direct *Pf* Ago-mediated cleavage of reporter probes, with no subsequent signal amplification. By comparison, *Pf* Ago functions only as a trigger to liberate active DNAzyme in the optimized system. Each released DNAzyme can cyclically cleave numerous fluorescent reporter substrates in the presence of Mg, thereby realizing efficient signal amplification. In addition, N. Wang et al. (2024a) utilized a centrifuge-based microfluidic chip to detect *S. aureus* (Figure 3B) (Zhao et al., 2024c). Previously, multiplex detection primarily depended on various fluorescent signals distinguished by color; but this method has obvious limitations-color overlap among multiple fluorophores can cause signal interference, affecting detection accuracy. In contrast, microfluidic chips possess multi-channel capabilities, enabling spatial differentiation of signals for different targets, thus achieving simultaneous multiplex detection and effectively addressing the limitations of traditional methods (Xie et al., 2022; Chen et al., 2023b). They developed an ASAP centrifuge microfluidic chip that integrate LAMP and *Tt*Ago systems, capable of detecting 16 samples simultaneously, thereby reducing reagent consumption and contamination risk. Initially, the sample under test is mixed with a nucleic acid rapid extraction reagent and injected into the CMC sample chamber, then centrifuged downward into the reaction chamber. The reaction chamber is pre-loaded with LAMP and *Tt*Ago systems. Consequently, species-specific *nuc* genes are amplified via LAMP, followed by *Tt*Ago-mediated site-specific cleavage, resulting in the emission of fluorescence signals. The LOD is 1 CFU/ml. Microfluidic chips enable the miniaturization of reaction systems, confining reagents and target nucleic acids within microscale chambers to elevate the local effective concentration of targets. Meanwhile, the chip integrates the entire workflow of nucleic acid extraction, amplification and *Tt*Ago-mediated cleavage-based detection, reducing nucleic acid sample loss and mitigating contamination risks. Coupled with the enzymatic signal-amplification effect, a lower limit of detection can therefore be achieved. Lateral flow immunoassay (LFA), as a paper-based on-site biosensing technique, offers significant advantages such as low cost, ease of operation, portability, rapid detection, and broad application scope, making it widely used in POCT (Hou et al., 2025). Qiao et al. (2024) developed a lateral flow biosensor (LFB) driven by *Pf* Ago for *Salmonella Typhimurium* detection, designated as *Pf* Ago LFB. They modified ssDNA reporter genes with FAM-biotin, enabling the generation of a colorimetric signal on the lateral flow strip. In negative samples, AuNP-anti-FAM antibodies conjugated with FAM-ssDNA-biotin reporter genes, which are intercepted by biotin ligands on the control strip, resulting in a red signal due to colloidal gold aggregation. This process requires only 45 min from sampling to results, with a LOD as low as 1 CFU/ml. Subsequently, they further innovated by implementing dual-mode colorimetric/fluorescent output, thereby enhancing the sensitivity and reliability of the detection system. As shown in Figure 3C, they combined *Pf* Ago with nucleic acid-based reverse-enhanced fluorescent lateral flow test strips (rLFTS) and smartphones, enabling ultrasensitive and portable detection of foodborne pathogens via cross-validation (Tang et al., 2025). *Pf* Ago recognizes LAMP products, triggers linker DNA cleavage, and generates dual colorimetric and fluorescent signals on the rLFTS. Specifically, when the target gene is present, the adapters 1DNA and 2DNA are selectively cleaved by *Pf* Ago, preventing the AuNPs@DNA probes from hybridizing to the capture probes (T1-BiotinDNA/R6G and T2-BiotinDNA/R6G), resulting in the absence of color development in the T1 and T2 lines, while R6G fluorescence remains intact. The methods enable the detection of Listeria monocytogenes virulence genes *hly* and *lde* via T1 and T2, respectively. Simultaneously, the AuNPs@DNA probes hybridize with the C-Biotin DNA/R6G capture probes at the control line, producing a red band indicative of AuNPs aggregation. Utilizing a 3D-printed imaging device and a smartphone application, pathogenicity and antibiotic resistance genes of Listeria monocytogenes can be detected within 45 min from sample to result, with a minimum detection limit of 1 CFU/ml. This platform offers a highly sensitive and portable approach for on-site multiplex detection of foodborne pathogens.

Integrating nanozymes as signal probes into the LAMP-*Pf* Ago system enhances the sensitivity of biosensors. As shown in Figure 3D, Zhang et al. (2025b) synthesized a nanomzyme comprising hemoglobin chloride immobilized within ZIF-90, exhibiting peroxidase-like activity. In the absence of target DNA, the linker ssDNA remains complete. In the presence of target DNA, the *invA* gene amplicon is specifically recognized and cleaved by *Pf* Ago. The resulting ssDNA then serves as a new guide DNA to activate *Pf* Ago's secondary cleavage activity, which cleavages the linker ssDNA substrate. Consequently, the cleaved linker ssDNA prevents hybridization between the DNA hybridization probe (ssDNA-MB) and the ssDNA-hemin@ZIF-90 probe. Following magnetic separation, the chlorinated hemoglobin@ZIF-90 nanozyme probe is dispersed into the supernatant to catalyze the colorimetric reaction between TMB and H_2_O_2_, generating a blue hue that corresponds proportionally to the concentration of Salmonella. The LOD is 1 CFU/ml. It leverages the specificity of *Pf* Ago and the signal amplification capabilities of MOF nanoenzymes to enhance detection accuracy and sensitivity, thereby providing an innovative approach for the development of next-generation biosensors in food safety. DNAzymes are a class of single-stranded DNA molecules, exhibiting protease-like catalytic activity. Curtis's team isolated a novel DNAzyme, designated Aurora, through *in vitro* selection (Volek et al., 2024). Zhang's team integrated *Pf* Ago with Aurora DNAzyme, which possesses both biosensing recognition elements and signal amplification capabilities, enabling highly sensitive and specific detection of foodborne pathogens (Zhang et al., 2025a). They engineered an unmarked signal probe, termed engineered-Aurora (E-Aurora), by inserting a 16-nucleotide target sequence fragment between two highly conserved regions of the DNAzyme responsible for Aurora (corresponding to nucleotide positions 10 and 11). This design enables *Pf* Ago to specifically cleave the E-Aurora probe, which catalyzes the conversion of substrate 4-methyl-umbrella phosphate (4-MUP) into a fluorescent product, 4-MU, producing an unlabelled amplified fluorescence signal at approximately 4,555 ± nm. Concurrently, through strategic design of non-conserved nucleotide sequences within Aurora, *Pf* Ago can specifically cleave E-Aurora to achieve efficient signal transduction. In the presence of Salmonella Typhi, LAMP products are specifically recognized by *Pf* Ago-gDNA, thereby activating the *Pf* Ago system; the activated *Pf* Ago then disrupts the catalytic core of E-Aurora, preventing the dephosphorylation of 4-MUP and ultimately producing a fluorescence “off” signal, with *S. Typhi* concentration inversely proportional to fluorescence intensity. The detection limit reaches as low as 1 CFU/ml.

Subsequently, they developed a triple-layer amplification strategy called PASS for pathogen biosensing (Figure 3E) (Lu et al., 2025). This architecture employs a ternary amplification strategy to achieve ultrasensitive detection of pathogenic bacteria. By LAMP amplification targeting the specific *invA* gene-constituting the first signal amplification. *Pf* Ago recognizes and sequentially cleaves the amplified products. To facilitate signal transduction, designed connectors couple AuNP-based SERS nanotags with magnetic particles (MPs), converting nucleic acid signals into enhanced SERS responses, representing the second signal amplification. The presence of Salmonella typhimurium DNA triggers the cleavage of the connectors, preventing the interaction between magnetic particles and nanotags, resulting in a red supernatant with a strong SERS signal after magnetic separation. Subsequently, AgNPs@PVA are physically mixed with AuNP-SERS nanotags to form electromagnetic-enhanced magnetic nano-SERS probes (EBM nSERS probes). Through chemical and electromagnetic amplification, the 4-MBA reporter signal is further enhanced, achieving a threefold enhancement in detection sensitivity. The work organically integrates four techniques, including nucleic acid isothermal amplification, sequence-specific cleavage mediated by prokaryotic Ago proteins, plasmonic enhancement of noble-metal-based SERS, and magnetic-separation-enabled background suppression, to construct a multi-level cascade signal-amplification system. Rather than relying on a single signal-enhancement strategy, it achieves outstanding detection sensitivity, enabling the detection of *S. Typhimurium* down to 1 CFU/ml. It fully compatible with handheld Raman devices, establishing a novel molecular diagnostic paradigm for on-site detection of foodborne pathogens.

### 3.2 Argonaute-based amplification-free detection for food safety

The previously described detection methods involve complex DNA extraction and amplification processes, which present significant disadvantages. Amplification requires specific temperature-controlled equipment and reaction times ranging from 30 min to several hours, which, combined with the 1–2 h needed for extraction, substantially extend the overall testing cycle. This prolongation hampers the timeliness essential for epidemic prevention and control, and the intricate procedures increase the risk of sample contamination and result instability. To address this, a new class of detection methods has been developed that eliminates the DNA extraction and amplification steps, reducing the sample preparation time from hours to minutes and lowering the requirements for conditions and personnel. (In this review, the term “amplification-free” is specifically defined as the absence of pre-amplification of the original target nucleic acid, such as PCR, LAMP and RPA. It does not indicate the total elimination of all signal-amplification processes.) Simplifying the process also reduces the risks of contamination and non-specific amplification, ensuring more accurate results and better meeting the practical demands of field testing.

#### 3.2.1 Argonaute-based amplification-free detection for bacteria

##### 3.2.1.1 Bacteriophages-based Argonaute amplification-free detection

**Figure 4:**
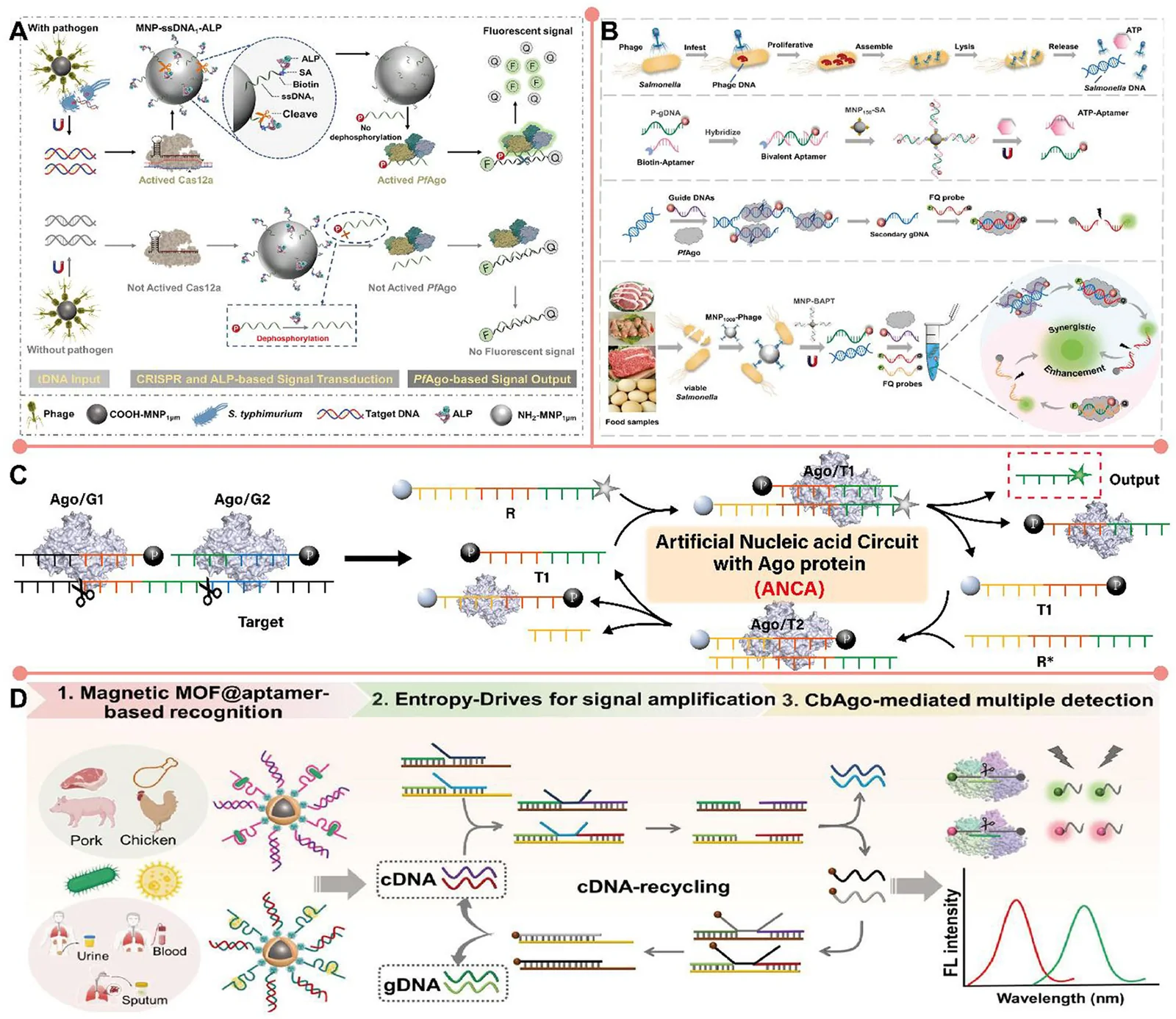
*Pf* Ago-based amplification-free detection for pathogenic bacteria. **(A)** CRISPR/Cas12a and Argonaute-mediated cascade signal amplification (Chen et al., 2023a). **(B)** DNA and ATP synergistically triggered Argonaute-mediated sensor (Wang et al., 2024b). **(C)** Argonaute-based one-step isothermal detection of CPKP (Jang et al., 2023). **(D)** Magnetic Fe_3_O_4_@MOF@aptamer-mediated entropy-driven fluorescence biosensor (Lu et al., 2024).

Bacteriophages, as a class of viruses distributed in natural environments, are extensively utilized in the detection of foodborne pathogens due to their excellent safety profile, high target specificity, and low manufacturing costs. Their primary mechanism involves specifically recognizing and infecting live host bacteria, injecting their genetic material into the bacterial cells, and utilizing the host's metabolic system to propagate progeny phages (Wan et al., 2022). This process leads to host cell lysis and the release of cellular components such as genomic DNA. Furthermore, the high specificity of bacteriophages for particular strains enables effective isolation of target pathogens from complex food matrices, substantially reducing the likelihood of false-positive results during detection (Tian et al., 2021). Yiping Chen and colleagues have proposed two novel methodologies leveraging bacteriophages as targeted nucleic acid extraction tools. These methods can specifically recognize and lyse viable bacterial cells, facilitating the release of target and obviating the requirement for conventional DNA extraction steps. The first approach is a nucleic acid detection platform based on a dual-enzyme cascade signal amplification (DECA) system mediated by CRISPR/Cas12a and *Pf* Ago (Chen et al., 2023a). As shown in Figure 4A, biotin-labeled ssDNA 1 binds to magnetic nanoparticles (MNPs). Through the biotin-streptavidin interaction, the modified MNPs are further conjugated with streptavidin-labeled alkaline phosphatase (SA-ALP). Due to ALP's high catalytic efficiency in dephosphorylation, a substantial amount of ssDNA 1-ALP conjugates can be ultimately attached to the MNP surface. Unrestricted ssDNA 1 exhibits Cas12a's non-specific single-strand DNase activity, cleaving ssDNA 1 and reducing the number of ALP molecules on the MNP surface post-magnetic separation. The residual ALP can only partially dephosphorylate the guide DNA (P-gDNA), resulting in insufficient dephosphorylation to activate *Pf* Ago cleavage activity. Since the initial concentration of P-gDNA directly influences the amount of *Pf* Ago capable of cleaving fluorescent reporter molecules, the fluorescence intensity correlates positively with the concentration of viable *S. Typhimurium*. The second approach is a PDAAF (Phage-Assisted DNA and ATP Co-triggered Argonaute-mediated Fluorescence) platform designed for highly sensitive detection of live *Salmonella* (Wang et al., 2024b). As shown in Figure 4B, when samples contain *Salmonella*, pre-assembled MNP-phage complexes specifically recognize and capture the target bacteria, followed by magnetic separation for effective enrichment and isolation. The phages only lyse live bacteria, releasing intracellular DNA and ATP. In the presence of ATP, the pre-fixed MNP-BAPT complex preferentially binds ATP, releasing its cargo of gDNA. After magnetic separation, the supernatant containing released gDNA assembles with *Pf* Ago to form a complex. This complex specifically recognizes and cleaves FQ probes, thereby generating an initial fluorescent signal. To further enhance detection sensitivity, researchers introduced three gDNA chains targeting Salmonella-specific DNA sequences. Fluorescence signals were amplified through a two-step FQ probe cleavage reaction, enabling the ultrahigh-sensitivity detection of live *Salmonella*. The minimum detection limits for these two methods are 23 CFU/ml and 20 CFU/ml, respectively, both demonstrating high accuracy and sensitivity.

##### 3.2.1.2 Nucleic acid circuit cascade-based Argonaute amplification-free detection

As illustrated in Figure 4C, Jang et al. (2023) reported an Argonaute-mediated self-catalytic nucleic acid detection system called ANCA (artificial nucleic acid circuit with Argonaute. Previous studies had developed various catalytic nucleic acid circuits, including polymerase/exonuclease/nuclease DNA loops, self-catalytic or cross-catalytic nucleic acid systems, hybridization chain reactions, and hairpin assembly. There had been no reports of constructing nucleic acid circuits using Ago proteins. ANCA is a fully DNA and Argonaute-based positive feedback loop that combines artificial nucleic acid circuits with the programmable cleavage activity of Argonaute. It successfully detects carbapenemase-producing *Klebsiella pneumoniae* with 100% sensitivity and specificity. In this system, the Argonaute forms complexes with G1 and G2, recognizing and hybridizing with the target DNA, followed by cleaving the target between the 10th and 11th nucleotides of the guide DNA, producing a short fragment T1. T1 then serves as a guide to form an AGO/T1 complex, which cleaves R to release a fluorescent output along with T2. Subsequently, T2 binds to Ago, forming a complex that cleaves R^*^, generating T1 and completing the DNA cycle. This positive feedback loop triggers repeated cleavage reactions, enabling the detection of target DNA in the sample by monitoring the fluorescence signal. Notably, the ANCA method relies on a single protein, simplifying manufacturing and reducing costs compared to conventional nucleic acid detection circuits. Yiping Chen first reported an entropy-driven fluorescent biosensor based on magnetic Fe_3_O_4_@MOF, termed the MAEA biosensor (Figure 4D) (Lu et al., 2024). Unlike ANCA, MAEA is a catalytic nucleic acid circuit centered on entropy-driven nucleic acid strand displacement. It requires the integration of metal-organic framework (MOF) magnetic carriers and *Cb*Ago-mediated signal readout, relying on multiple auxiliary components for detection. Due to the strong coordination of Zr-O-P bonds, aptamers can stably conjugate onto the surface of magnetic Zr-based MOF (Fe_3_O_4_/SiO_2_/UiO66), forming Fe_3_O_4_/SiO_2_/UiO66/Apt complexes. Pathogenic bacteria and cDNA competitively recognize the aptamers on the complex surface. The aptamers exhibit a higher affinity for pathogens than for cDNA, leading to hybridization when pathogens are absent and dissociation of cDNA in solution when pathogens are present. Magnetic separation isolates the complexes, with the supernatant cDNA content proportional to pathogen load. Subsequently, the resulting supernatant is subjected to EDC-mediated signal amplification. In this process, a three-stranded DNA complex (Q/P/gDNA) is formed by Q, P and gDNA-Q, which contains a single-stranded foothold structure. This complex enables the binding of target cDNA, accompanied by the release of P strand and the formation of stable Q/gDNA/cDNA complexes. Subsequently, a biotinylated fuel strand (F) binds to the complex, releasing gDNA and cDNA, which triggers a new cycle and generates abundant gDNA related to the target via an EDC reaction. The F strand is labeled with biotin, and after the EDC reaction, streptavidin-modified 150 nm magnetic nanoparticles (MNP150-SA) remove Q/F complexes, preventing interference in subsequent *Cb*Ago cleavage. Finally, the pathogen-specific gDNA is assembled with *Cb*Ago guided by the corresponding DNA, enabling precise and efficient cleavage of reporter molecules labeled with different fluorogenic quenchers, producing specific fluorescence signals. This allows the MAEA biosensor to detect *S. aureus* and *E. coli* with high sensitivity (< 10^2^ CFU/ml).

##### 3.2.1.3 Digital-based Argonaute amplification-free detection

**Figure 5:**
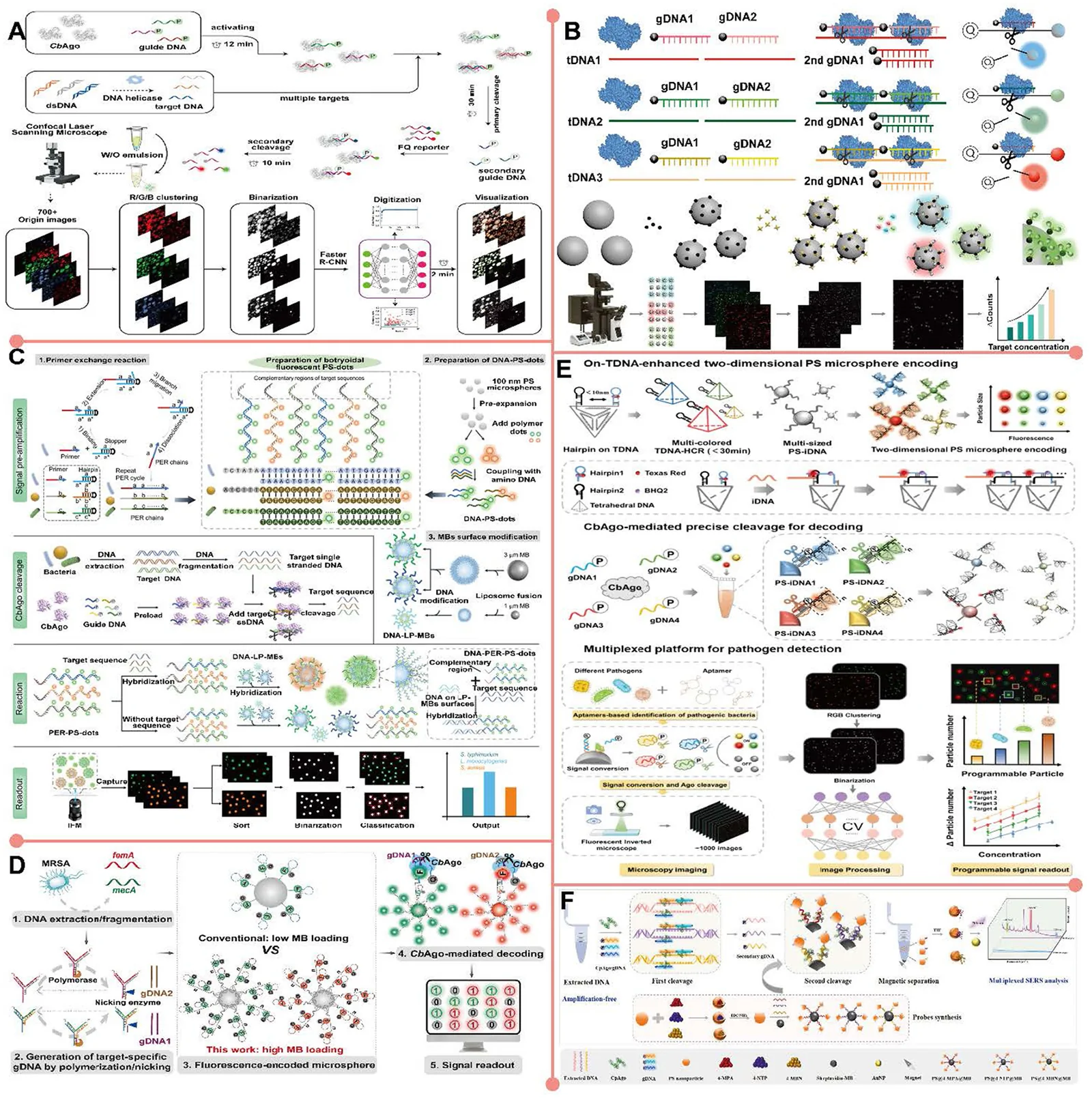
*Cb*Ago-based amplification-free detection for pathogenic bacteria. **(A)** Deep learning-based multi-vessel microdroplet signal readout system for nucleic acid detection using *Cb*Ago (Wang et al., 2024c). **(B)** Argonaute-mediated digital sensor based on a magnetic-bead-assisted imaging transcoding system (Wang et al., 2025b). **(C)** An amplification-free digital assay based on primer exchange reaction-mediated botryoidal-like fluorescent polystyrene dots (Wang et al., 2024d). **(D)** Detection of MRSA via fluorescence-encoded microsphere and Argonaute-mediated decoding (Wei et al., 2024). **(E)** Multiplexed pathogenic bacteria detection via a two-dimensional encoded fluorescent microsphere system (Wang et al., 2025a). **(F)** Programmable mesophilic Argonaute-triggered SERS biosensor for amplification-free and multiplexed nucleic (Liu et al., 2025).

Digital analysis is a technique based on the segmentation of reaction systems. The entire system is partitioned into numerous microreactor units, with the outcomes of each microreaction statistically analyzed during the final processing stage (Li et al., 2023a). One method for producing droplets involves immersing water in oil. As shown in Figure 5A, the team led by Chen utilized a *Cb*Ago-mediated PASS system to achieve simultaneous detection of multiple foodborne pathogen DNAs, with a detection limit reaching less than 10^2^ CFU/ml (Wang et al., 2024c). The PASS technology benefits from multi-dispersed emulsion techniques combined with a multilayered microdroplet artificial intelligence readout system developed in this study. Firstly, the *Cb*Ago protein complexed with guide DNA-a deoxyribonucleoprotein complex (DRNPs), can cleave DNA targets without prior linearization, generating DNA fragments that serve as new guides for the cleavage of fluorogenic quencher (FQ)-labeled probes. *Cb*Ago directly opens double-stranded plasmids to create nicks, while under the assistance of Escherichia coli RecQ helicase, it further linearizes partially opened plasmids. These topologically modified plasmids, resulting from primary DRNP-mediated cleavage, can then activate secondary cleavage reactions. Subsequently, the entire *Cb*Ago catalytic reaction system is emulsified via physical emulsification in a continuous phase composed of corn oil and surfactants. Confocal laser scanning microscopy (CLSM) is used to image the emulsion, and the acquired images are processed through deep learning algorithms to quantitatively analyze fluorescence intensity. Similarly, Yiping Chen's team proposed a digital nucleic acid detection method called d-MAGIC (mesophilic *Cb*Ago and magnetic beads in a digital carrier system; Figure 5B) (Wang et al., 2025b). This method first designs specific gDNA for *S. typhimurium, S. aureus*, and *L. monocytogenes*, with 5′-phosphorylation modifications. After hybridization with pathogen DNA, *Cb*Ago cleaves at designated sites, generating secondary gDNA. This secondary gDNA subsequently forms novel complexes with *Cb*Ago, facilitating the binding and cleavage of biotin-fluorescent end-labeled FQ reporters. Simultaneously, the method substitutes the monodisperse droplets utilized in conventional digital nucleic acid analysis with uniform MBS for AI-based decoding of digital counts. Carboxyl-modified MBs are grafted with tyramine and covalently linked with streptavidin to prepare MB-TA-SA conjugates. The biotin with fluorescent labels interacts with streptavidin-biotin to encode three distinct fluorescent signals on the conjugate surface for digital readout. Subsequently, substrates are removed via magnetic separation, and confocal laser scanning microscopy (CLSM) captures fluorescence images of the encoded MBs. These images are converted into grayscale and decoded by AI, establishing a quantitative relationship between the encoded MBs and target DNA. This facilitates the digital and multiplex detection of three foodborne pathogens without the need for DNA amplification. The detection limit of d-MAGIC is as low as 6 CFU/ ml. D-MAGIC emerges as a promising next-generation technology that combines precision with convenience for pathogen detection. Furthermore, they implemented an AI-based fluorescence microsphere counting algorithm to achieve digital quantification. A digital detection method was developed based on DNA-PER-modified grape-like PS dots (DNA-PER-PS-dots; Figure 5C) (Wang et al., 2024d). First, the preprocessing of the digital signal probes is performed by sequentially preparing DNA single strands, DNA-PS-dots, and DNA-LP-MBS constructs. The pseudoknot region concurrently displays the hybridization of the DNA-PER strands with DNA-PS-dots, resulting in grape-like fluorescent aggregation points (PER-PS-dots). Subsequently, *Cb*Ago mediates the site-specific cleavage reaction. Following this, fluorescence encoding is achieved through assembly, where the cleaved target sequences serve as bridging elements: one end hybridizes with the surface DNA of DNA-LP-MBS via complementary base pairing, while the other end hybridizes with the DNA strands of PER-PS-dots. Thus, completing the specific fluorescent coding of DNA-LP-MBS. Finally, after fluorescence microsphere imaging and digital image processing, AI algorithms are employed to identify positive signals and perform digital readout. Their group has also pioneered the development of a fluorescent biosensor. This sensor employs tetrahedral DNA (TDNA)-modified polystyrene (PS) microspheres as fluorescence-encoding carriers and integrates *Cb*Ago -mediated decoding strategy, enabling ultra-sensitive detection of MRSA *mecA* and *femA* genes without pre-amplification (Figure 5D) (Wei et al., 2024). The detection principle involves target proximity hybridization-induced polymerization and nicking cycles that generate guide DNA, which directs *Cb*Ago to specifically cleave molecular beacons on the microspheres, thereby decoding fluorescence signals. Simultaneously, the microspheres utilize RCA amplification on the tetrahedral DNA scaffold to improve the loading efficiency of molecular beacon (MB) probes. This substantially elevates the fluorescence response and facilitates trace-level target detection, with the limits of detection reaching as low as 0.63 fM for *mecA* and 0.48 fM for *femA*, respectively. A programmable microscopic imaging platform has been developed, integrating ultra-bright two-dimensional coded fluorescent microspheres with a *Cb*Ago-mediated signal decoding system to achieve highly sensitive, amplification-free detection of pathogenic bacteria (Figure 5E) (Wang et al., 2025a). Initially, a multi-signal probe was constructed using tetrahedral DNA-enhanced hybridization chain reaction (TDNA-HCR), resulting in the synthesis of ultra-bright polystyrene microspheres (PS-TDNA-HCR) labeled with multiple fluorophores. The rigid TDNA structure stabilizes the hairpin probes and optimizes fluorophore density, significantly improving reaction efficiency. Subsequently, the PS-TDNA-HCR probes are two-dimensionally encoded based on fluorophore color and particle size. During detection, pathogen information is converted into gDNA via an aptamer-based recognition mechanism, activating the *Cb*Ago to cleave the initial DNA bridge, thereby releasing fluorophores from the PS-TDNA-HCR surface to amplify the signal. Concurrently, a computer vision -based algorithm classifies the fluorescent carriers by color, size, and count, enabling the simultaneous detection of up to four pathogenic bacteria. This study represents the first report of a programmable microscopic imaging platform centered on tetrahedral HCR-assisted fluorescent probe encoding and *Cb*Ago-mediated decoding, offering a novel strategy for ultra-sensitive detection of multiple pathogens.

##### 3.2.1.4 SERS-based Argonaute amplification-free detection

In addition to using fluorescence output signals, SERS has gained increasing popularity for signal detection due to its strong anti-interference capability, ease of operation, high sensitivity at the single-molecule level, and its rich yet narrow spectral fingerprint. SERS can overcome the limitations of background interference and restricted fluorescent probe diversity inherent in traditional fluorescence-based multiplex detection methods (Xu et al., 2019; Jiang et al., 2021; Yuan et al., 2021). A novel programmable mesophilic Clostridium perfringens Argonaute (*Cp*Ago)-triggered SERS sensing platform (*Cp*Ago-SERS) was proposed (Figure 5F). In this system, dual rounds of continuous cleavage mediated by *Cp*Ago/gDNA complex ensure specificity in DNA recognition and effective signal amplification. To further enhance detection sensitivity, Raman report molecules are encapsulated within controllably loaded polystyrene (PS) nanoparticles. These nanoparticles efficiently load signaling molecules, thereby achieving additional signal amplification following target activation-induced *Cp*Ago cleavage. Remarkably, this platform allows multiplex nucleic acid detection within a single vessel without any pre-amplification steps, achieving a detection limit as low as 0.497 ng/μl (Liu et al., 2025).

#### 3.2.2 Amplification-free Argonaute for toxin

**Figure 6:**
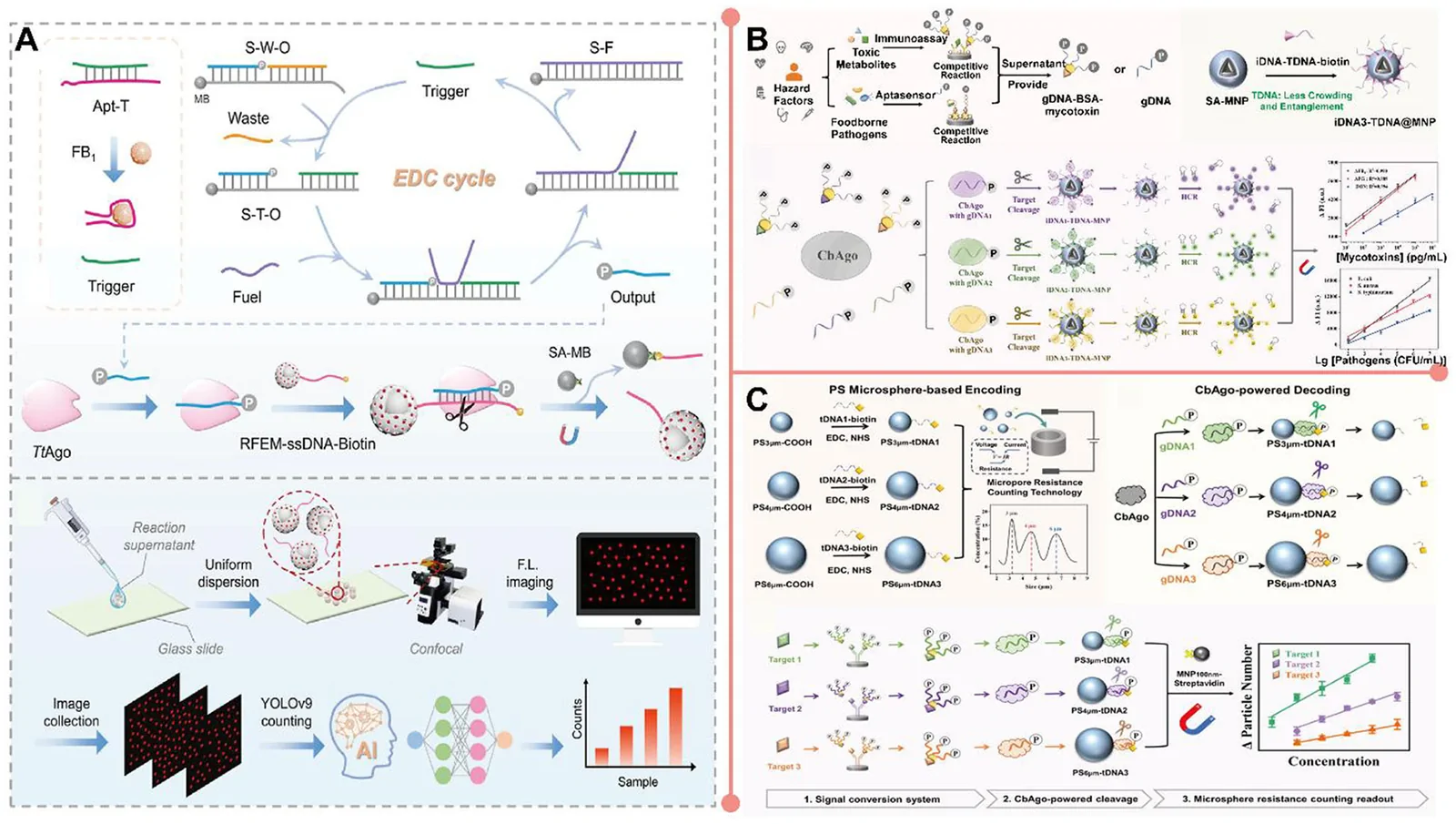
Argonaute-based amplification-free detection for mycotoxin. **(A)** Argonaute and entropy-driven catalysis Fumonisin B1 detection (Lin et al., 2025). **(B)** *Cb*Ago-based magnetic nanoparticle tetrahedral DNA encoding system (Li et al., 2024a). **(C)** Micropore resistance counting platform for multiplexed detection of Mycotoxins and biomarkers (Li et al., 2025b).

*Fumonisin* B1 (FB1) is a prevalent mycotoxin primarily produced by Fusarium verticillioides, frequently contaminating corn and corn-based products. As shown in Figure 6A, Wu's team has developed an ultra-sensitive detection platform for FB1 based on deep learning and entropy-driven catalysis (EDC)-Argonaute technology, utilizing a fluorescent single-particle assay. This platform employs biotinylated single-stranded DNA and red fluorescent-coded microspheres as signal probes, with streptavidin-labeled magnetic beads serving as separation carriers (Lin et al., 2025). Specifically, upon binding with FB1, the aptamer releases a trigger sequence that mediates EDC cycling to generate a large amount of 5′-phosphorylated output sequences. These sequences act as guide DNA to activate *Tt*Ago, cleaving the signal probes and increasing the number of residual fluorescent microspheres in the supernatant after magnetic separation. Subsequently, YOLOv9 deep learning model rapidly and accurately counts the red fluorescent particles in confocal fluorescence images of the supernatant. The method achieves a detection limit as low as 0.89 pg/ml, with a linear range from 1 pg/ml to 100 ng/ml. This provides robust support for the application of single-particle fluorescence detection technology in food safety monitoring.

Yiping Chen et al. innovatively constructed a *Cb*Ago-powered programmable sensing platform. Based on tetrahedral DNA (TDNA) and magnetic nanoparticle (MNP) encoding systems, this platform enables multiplexed quantification of non-nucleic acid targets at 37 °C (Figure 6B) (Li et al., 2024a). This platform achieves ultrahigh sensitivity via two synergistic mechanisms. First, DNA tetrahedral structures are adopted to improve structural stability and mitigate interference between proteins and nucleic acid probes. Second, enzyme-free hybridization chain reaction (HCR) is initiated at the vertices of DNA tetrahedra, thereby greatly elevating the overall detection sensitivity. In practical sample analysis, this platform has been successfully applied for the multiplex detection of three mycotoxins in 30 food samples (detection level: pg/ml) and three pathogenic strains, including *Escherichia coli, Staphylococcus aureus*, and *Salmonella Typhimurium*, in 100 clinical samples (detection level: 10^2^ CFU/ml), all of which exhibited desirable and reliable detection performance. Subsequently, they further constructed a system based on drug resistance counting using modified polystyrene microspheres encoded with target DNA (Figure 6C) (Li et al., 2025b). This system leverages DNA-guided *Cb*Ago protein to precisely recognize and cleave the encoded microspheres, decoding the system based on changes in polystyrene microsphere concentration as the signal. In practical detection, this platform demonstrated excellent performance, enabling multiplex detection of three mycotoxins (Aflatoxin B1, Ochratoxin A, Deoxynivalenol) with sensitivity spanning over four orders of magnitude at pg/ml levels, as well as two inflammation markers (procalcitonin PCT, interleukin-6 IL-6) at pg/ml levels.

## 4 Challenge and prospects

### 4.1 Challenges of Argonaute-based detection

**Figure 7:**
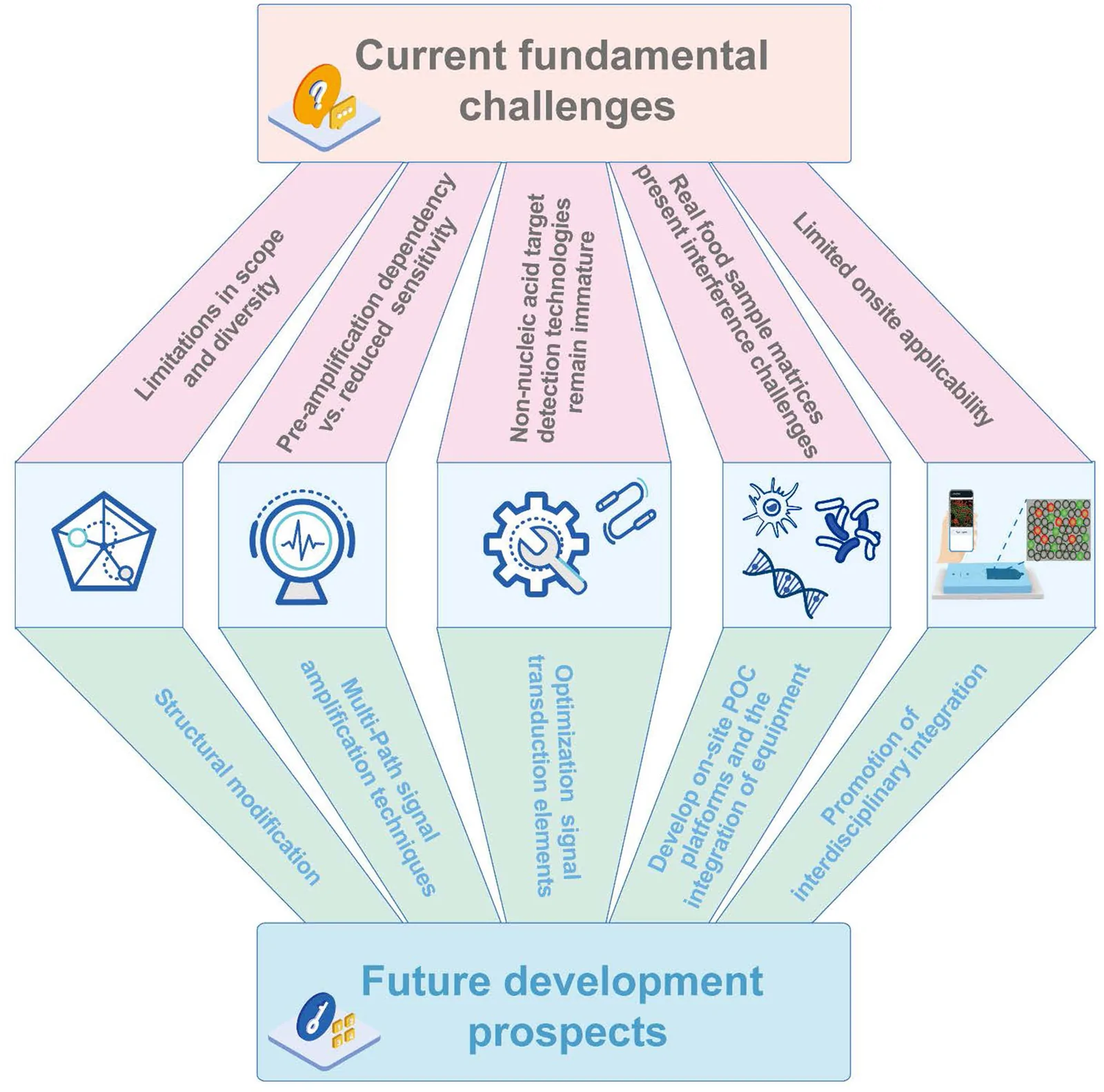
The current fundamental challenges and future development prospects.

Argonaute-based detection technologies have exhibited precise nucleic acid recognition, efficient cleavage activity, and programmability in the field of food safety detection. Currently, thermophilic Argonaute (e.g., *Tt*Ago, *Pf* Ago) are the pre-dominant choice for applications, while research on mesophilic Argonaute (e.g., *Cp*Ago, *Cb*Ago) is gradually advancing. However, the transition of this technology from laboratory research to practical application still faces challenges (Figure 7): (1) There are inherent limitations in the performance of Argonaute. Thermophilic Argonaute requires maintaining optimal nuclease activity at 66–95 °C, rendering it unsuitable for POCT. Although mesophilic Argonaute can operate at lower temperatures. Their tighter crystalline structures restrict molecular interactions, resulting in significantly reduced cleavage activity compared to thermophilic Argonaute. Thus, additional signal amplification techniques are necessitated, which are complex and hinder widespread adoption. (2) There is a conflict between the reliance on pre-amplification and the sensitivity of amplification-free detection. Most Argonaute-based detection assays depend on pre-amplification steps such as PCR, LAMP, or RPA for enhancing sensitivity. However, amplification processes face obstacles, including the need for bulky thermal cyclers, non-specific amplification, and risks of aerosol contamination. Conversely, amplification-free detection can avoid these problems by combining advanced technologies such as nano-materials and SERS, but often greatly reduces sensitivity. It remains a critical challenge to improve the LOD of amplification-free detection. (3) The technology for non-nucleic acid target detection remains immature. Argonaute systems are naturally suited for nucleic acid targets. Nevertheless, the application to non-nucleic acid targets like fungal toxins, ATP is still in the early stages. The primary obstacle is the lack of an effective signal transduction bridge between non-nucleic acid targets and Argonaute. (4) Complex components in real food samples-such as carbohydrates, proteins, lipids, and ions-significantly interfere with detection sensitivity and accuracy. Most Argonaute-based detection methods have been validated only in standard buffer solutions, without fully considering matrix effects. (5) In terms of field application and multiplexed detection, existing methods are primarily validated in laboratory settings, relying on bulky instruments like fluorescence detectors and requiring specialized personnel. Although colorimetric LFA has been widely exploited to construct point-of-care (POC) sensing platforms, the inherent limitations of biological receptors severely restrict their analytical performance, inevitably leading to inferior sensitivity and high false-positive rates.

### 4.2 Optimization and prospects for Argonaute-based detection

Despite encountering the challenges, Argonaute has exhibited exceptionally wide-ranging and promising development prospects: (1) For Argonaute performance optimization, one approach is altering the structure of mesophilic pAgos via introducing genetic modification and protein engineering techniques. This approach can overcome the constraints imposed by crystal structures on molecular interactions, yielding variants with high cleavage activity and fundamentally addressing the issue of low activity at moderate temperatures. Another approach is the discovery of novel pAgos with superior properties. *Mbp*Ago, from the cold adapted bacterium *Mucilaginibacter paludis*, maintains activity over a wide temperature range of 4–65 °C (Li et al., 2022b). *Mbp*Ago has superior RNA cleavage activity compared with DNA. Besides, there was no 5′ nucleotides preference, which can significantly reduce detection complexity and cost, further enriching the available detection toolkit. (2) To overcome the sensitivity bottleneck of amplification-free detection, multiple innovative approaches have been employed, such as the integration with SERS, graphene field-effect transistors, and nanopore sensing platforms, which could leverage the signal amplification effects of nanomaterials for ultra-sensitive detection (Li et al., 2025d). Most strikingly, the construction of nucleic acid circuits or cascade signal amplification strategies can mechanistically amplify signals to further improve the LOD. (3) For non-nucleic acid targets detection, transcription factors, like aptamer, DNAzymes, antigen-antibody interactions, and riboswitches can be the direction of research (Dong et al., 2025). Although pesticide and veterinary drug residues pose severe threats to food safety, studies on Argonaute-based sensing for these small-molecule non-nucleic-acid analytes remain limited at present. Nevertheless, aptamer-facilitated molecular sensing provides a feasible route to translate small-molecule recognition into nucleic-acid-readable signals. Benefiting from the modular characteristics of Ago-based detection systems, future efforts can couple high-performance aptamer recognition elements with Ago signal transduction, which holds great potential for agricultural residue monitoring in real-world food samples. (4) To advance POC platforms and device integration, sample pre-processing steps like microneedle extraction and microfibril filtration can be introduced. Argonaute-mediated cleavage reactions and signal readout can be integrated into microfluidic chips, nanopore arrays, or microchamber arrays to realize automated “sample input-result output” workflows, which can reduce manual operation errors and cross-contamination risks. CRISPR/Cas-based on-site detection can be referenced, which guide Argonaute to couple with smartphones for reading fluorescence or colorimetric signals. (5) Furthermore, the integration of big data, cloud computing, and artificial intelligence is also crucial. For example, reaction conditions (e.g., temperature, divalent ion concentration, gDNA parameters) can be optimized through machine learning to enhance cleavage efficiency and accuracy (Zhao et al., 2024a). Machine learning-assisted result interpretation can improve the practicality of POC applications.

Overall, the challenges associated with Argonaute-based detection primarily concern protein performance, sensitivity conflicts, non-nucleic acid detection, and adaptability for on-site applications. By optimizing proteins, innovating amplification-free techniques, integrating devices, and fostering multidisciplinary collaboration, it is poised to overcome current limitations. Argonaute holds the potential to become a versatile tool in food safety testing and molecular diagnostics, driving the industry toward greater sensitivity, speed, and portability.

## 5 Conclusions

This review consolidates the fundamental biochemical principles of Argonaute and highlighted recent advancements in Argonaute-based detection within the field of food safety. By highlighting key achievements, it aids researchers in gaining a deeper understanding of current strategies for constructing detection assays, thereby addressing the existing knowledge gap. In summary, as an emerging and fast-evolving analytical technology, the Argonaute system exhibits remarkable application potential in food safety monitoring and human health protection, holding great promise as an ideal alternative to conventional analytical methodologies. We anticipate that Argonaute-based detection technologies will continue to advance, ultimately evolving toward predictive, interactive, traceable, and multifunctional capabilities. Such developments will drive progress in molecular diagnostics, enabling more efficient, comprehensive, and intelligent approaches to safeguarding biological safety. We eagerly look forward to the transition of Argonaute technology from laboratory research to practical applications, with the expectation that its advantages can be effectively realized beyond controlled experimental settings.
